# Detection of physiological deterioration by the SNAP40 wearable device compared to standard monitoring devices in the emergency department: the SNAP40-ED study

**DOI:** 10.1186/s41512-018-0040-7

**Published:** 2018-09-03

**Authors:** Matthew J. Reed, Megan McGrath, Polly L. Black, Steff Lewis, Christopher McCann, Stewart Whiting, Rachel O’Brien, Alison Grant, Beth Harrison, Laura Skyrme, Miranda Odam

**Affiliations:** 1 Emergency Medicine Research Group Edinburgh (EMERGE), Department of Emergency Medicine, Royal Infirmary of Edinburgh, 51 Little France Crescent, Edinburgh, EH16 4SA UK; 2 Acute Care Group, Usher Institute of Population Health Sciences and Informatics, College of Medicine and Veterinary Medicine, University of Edinburgh, Nine Edinburgh BioQuarter, 9 Little France Road, Edinburgh, EH16 4UX UK; 3 snap40, 24 Forth Street, Edinburgh, EH1 3LH UK; 4 Edinburgh Clinical Trials Unit and Centre for Population Health Sciences, Usher Institute of Population Health Sciences and Informatics, Nine Edinburgh BioQuarter, 9 Little France Road, Edinburgh, EH16 4UX UK; 5 Emergency Department, Royal Infirmary of Edinburgh, 51 Little France Crescent, Edinburgh, EH16 4SA UK

**Keywords:** Ambulatory monitoring, Monitoring, physiological, Patient monitoring, Clinical deterioration

## Abstract

**Background:**

In recent years, there has been increasing focus on the earlier detection of deterioration in the clinical condition of hospital patients with the aim of instigating earlier treatment to reverse this deterioration and prevent adverse outcomes. This is especially important in the ED, a dynamic environment with large volumes of undifferentiated patients, which carries inherent patient risk. SNAP40 is an innovative medical-grade device that can be worn on the upper arm that continuously monitors patients’ vital signs including relative changes in systolic blood pressure, respiratory rate, heart rate, movement, blood oxygen saturation and temperature. It uses automated risk analysis to potentially allow clinical staff to easily and quickly identify high-risk patients. The aim of this study is to investigate whether the SNAP40 device is able to identify deterioration in the vital sign physiology of an ED patient earlier than current standard monitoring and observation charting techniques.

**Methods/design:**

Single centre, teaching hospital ED open label, prospective, observational cohort study recruiting 250 high acuity participants aged 16 years or over presenting to the ED. Participants will be approached and enrolled in the ED and after consent will have the SNAP40 wearable monitoring device attached which will be used alongside standard care monitoring. Participants will be observed throughout their time in the ED. Any SNAP40 device alarm, standard monitoring alarms or standard practice vital sign observations indicating a deterioration in a patient’s vital sign physiology (defined as an increase in NEWS score) will be recorded. Primary outcome is time to detection of deterioration. Secondary outcomes include staff time spent performing observations and responding to standard monitoring alarms, clinical escalation of care when deterioration is detected and participants and staff rating of experience of both SNAP40 and current monitoring.

**Discussion:**

The SNAP40-ED study aims to recruit 250 patients. It will be the first study to compare the ability of a novel ambulatory monitoring device to detect deterioration compared to standard care in the ED. It may allow the earlier detection of deterioration in the clinical condition of ED patients and therefore earlier treatment to reverse this deterioration and prevent adverse outcomes.

**Trial registration:**

NCT03179267 ClinicalTrials.gov. Registered on June 17, 2017

## Background

In the last decade, there has been increasing focus on the earlier detection of deterioration in the clinical condition of hospital patients with the aim of instigating earlier treatment to reverse this deterioration and prevent adverse outcomes. In studies of cardiac arrest, it was recognised that it was the final step in a series of events [1] that often went overlooked and once cardiac arrest occurred, prognosis was vastly reduced [2]. The NCEPOD 2012 report on in hospital cardiac arrest [3] showed 73% of patients had at least one marker of instability prior to cardiac arrest. In 62%, this was present 6 h prior to cardiac arrest, and in 47%, this was present 12 h before. This deterioration had not been recognised in 36% of cases, and in 38% of cases, the cardiac arrest could have been prevented altogether. These and many other studies led to an agreement that earlier recognition of physiological derangement may allow the possibility of improving patient outcomes. Focus turned firstly to techniques to improve the detection of physiological derangement, and secondly to strategies to allow these physiological derangements to be addressed once detected, for example with medical emergency teams (MET) [4].

The recognition of physiological derangement however is still problematic. The 2013 UK Parliamentary and Health Service Ombudsman report ‘Time to act—Severe sepsis: rapid diagnosis and treatment saves lives’ highlighted that the lack of early recognition and proper monitoring led to worse patient outcomes and increased costs [5]. Practice in the emergency department (ED) is seemingly no better with the 2011–2012 Royal College of Emergency Medicine audit report finding that the monitoring of vital signs in patients who went on to develop severe sepsis or septic shock was below standard and led to later recognition and poorer outcomes [6].

The availability of senior clinicians trained in managing the acute deteriorating patient is not as problematic in the ED as on hospital wards where solutions such as MET teams are required. The ED however is a dynamic environment with large volumes of undifferentiated patients, which carries inherent patient risk. EDs across the UK also now have increase demand, greater financial pressures, critical staffing issues and patient flow issues with exit block [7]. Recognition of deteriorating patients within the ED is a significant patient safety concern, and techniques to improve the detection of physiological derangement have included Early Warning Scores (EWS) such as National Early Warning Score (NEWS) [8]. The Royal College of Emergency Medicine and Royal College of Physicians support the use of the NEWS within EDs to improve recognition of deteriorating patients and escalation of physiological unstable patients to senior doctors [9, 10]. NEWS works on a scoring system (Table 1) that, along with clinical judgement, is used to inform a structured response.

**Table 1:** National Early Warning Score (NEWS) [10]

Physiological parameters	3	2	1	0	1	2	3
Respiration rate	≤ 8		9–11	12–20		21–24	≥ 25
Oxygen saturations	≤ 91	92–93	94–95	≥ 96			
Any supplemental Oxygen		Yes		No			
Temperature	≤ 35.0		35.1–36.0	36.1–38.0	38.1–39.0	≥ 39.1	
Systolic BP	≤ 90	91–100	101–110	111–219			≥ 220
Heart rate	≤ 40		41–50	51–90	91–110	111–130	≥ 131
Level of consciousness				A			V, P or U

Despite the introduction of EWS, early recognition of physiological instability remains an issue [5, 11–13]. Although it is clear that healthcare staff do ultimately recognise patients that are critically ill, crucial time is being lost [14] leading to increased patient length of stay, increased deaths and increased admissions to ICU [15]. The likely problem is that EWS rely on staff manually taking and documenting observations at set intervals which is problematic in a busy ED.

The recent ubiquitous rise in smart phones and internet access has led to a surge in applications which interconnect everyday objects with the internet (https://www.wired.com/2013/05/internet-of-things-2). Devices such as fitness devices and smart watches are commonplace with one in six consumers in the USA currently using wearable technology (https://www.forbes.com/sites/bernardmarr/2016/03/18/15-mind-boggling-facts-about-wearables-in-2016/#1b6706b27323). Wearable medical-grade devices (e.g. continuous blood glucose monitoring in diabetes, electrocardiogram (ECG) recording and falls prevention in the elderly have followed, and NHS England’s Five Year Forward document highlights the requirement for exploitation of the information revolution to accelerate health innovation [16].

SNAP40 is an innovative medical-grade device that can be worn on the upper arm. The device continuously monitors patients’ vital signs including relative changes in systolic blood pressure, respiratory rate, heart rate, movement, blood oxygen saturation and temperature. It uses automated risk analysis to potentially allow clinical staff to easily and quickly identify high-risk patients. It has the potential to reduce the time taken for staff to detect deterioration in patient physiology using NEWS score-based changes as well as other specific contributing physiological markers.

The scoring system is the same as with NEWS (Table 1) except for two parameters, blood pressure and movement. A score of ‘1’ is given if the blood pressure increases or decreases by 15% of the patient’s initial reading. Movement is recorded as either ‘normal’ or ‘reduced’. If movement is reduced, then a score of ‘1’ is given. As well as the potential of the SNAP40 device to improve patient safety, it may potentially increase ED efficiency, improve and increase use of resources and improve patient flow.

Currently, the only option for continuous monitoring or regular observations in the ED is to connect the patient to a standard monitor. Whilst these monitors can be portable, they are not lightweight enough to allow the patient to be ambulatory and therefore require the patient to have a bed and a bed space or cubicle. This leads to lack of space within the ED, less cubicles (which allow privacy) free for performing history taking, examination and procedures, difficulty accessing patients and difficulty transporting them to areas where specialist tests can be performed (i.e. radiology and ECG). SNAP40 may remove the need for patients to be confined to beds and bed spaces (unless clinically required) freeing up space within the ED and allowing the ED care processes to occur much more freely, increasing efficiency and speed of patient processing. This may lead to improved patient ED and hospital experience.

The SNAP40 device may also potentially free up resources (e.g. reducing time clinical staff record observations), which can be used to improve patient care and experience in the ED (e.g. timelier analgesia and treatments, more time for communication and traditional nursing and personal care).

There are some other similar medical devices on the market (i.e. www.biovotion.com, www.sensium-healthcare.com, www.vitalconnect.com, www.caretakermedical.net) using wristbands, patches or finger cuff technology, and whilst these devices may be suitable, none currently are being used in ED clinical practice. The SNAP40 device was chosen by the SBRI for study in the ED environment and is therefore the chosen device for our study.

This study will examine the SNAP40 device by comparing its performance to detect physiological deterioration against standard observations taken by clinical staff (nurses, doctors and clinical support workers) within the ED. The study will assess whether the device is able to detect physiological derangement sooner than standard monitoring devices.

### Study aims

#### Primary aim

The primary aim of the study is to investigate whether the SNAP40 device is able to identify deterioration in the vital sign physiology of an ED patient earlier than current standard monitoring and observation charting techniques.

#### Secondary aims

1. To determine whether the SNAP40 device can potentially reduce the time ED staff spend performing and charting observations and responding to standard monitoring alarms
2. To assess how often a change in the vital sign physiology of an ED patient leads to a clinical escalation or de-escalation of care
3. To study ED patient experience rating of both SNAP40 and current monitoring techniques and patient confidence with future SNAP40 device use alone
4. To study ED staff experience rating of both SNAP40 and current monitoring techniques and ED staff confidence with future SNAP40 device use alone

## Methods

### Design

This is a single centre, teaching hospital ED open label, prospective, observational cohort study.

### Setting

The setting is the ED of the Royal Infirmary of Edinburgh (RIE). The RIE sees approximately 120,000 patients per year of whom approximately half are deemed to be majors level acuity.

### Primary endpoint

Time to detection of deterioration in an ED patient’s vital sign physiology is defined as an increase in NEWS score. This would include a deterioration in blood pressure, pulse/heart rate, respiratory rate, skin temperature (SNAP40)/core temperature (standard), oxygen saturation reading or movement (SNAP40)/Glasgow Coma Scale (GCS) (standard).

### Secondary endpoints

1. Time ED staff spend performing a charting observations and responding to standard monitoring alarms
2. Percentage clinical escalation of care when deterioration detected
3. Participants rating of experience of both SNAP40 and current monitoring
4. Research nurse rating of experience of both SNAP40 and current monitoring

We have not performed power calculations for our secondary outcomes that are purely descriptive and for qualitative purposes. We aim to recruit 250 participants into the study. We will aim to recruit at least 50 participants who are triaged to a non-monitored area and at least 50 participants who are triaged to continuous monitoring. Other recruited participants will have variable levels of observation charting as determined by the treating clinical team. After surveying a group of ED and critical care physicians, we selected an increase in NEWS score of + 1 as being a clinically significant endpoint.

### Population

Two hundred fifty participants aged 16 years or over presenting to the ED of the RIE who are triaged to majors (high dependency or immediate care) or who are stepped down from resuscitation room care to high dependency or immediate care shall be recruited into the study. Recruitment will last around 4 months.

### Inclusion criteria

1. Participant aged 16 years or over
2. Participant triaged to majors (high dependency or immediate care)
3. Participants who are stepped down from resuscitation room care to high dependency or immediate care

### Exclusion criteria

1. Participants under 16 years of age
2. Previous participation in the study
3. Participant in custody
4. Participants deemed high risk for absconding by clinical staff
5. Participants unable to communicate in English
6. Participants who are triaged to immediate resuscitation. These participants may be considered for inclusion once immediate assessment and treatment have been initiated and they are stepped down to high dependency (HD)/immediate care (IC) areas
7. Patients with implantable defibrillators, pacemakers or neurostimulators will be excluded
8. Patients who cannot have blood pressure measured in both arms, e.g. patients with renal fistula and a peripherally inserted central catheter (PICC) line, or who have had a lymph node clearance

n.b. Scotland A Research Ethics Committee approved inclusion of participants lacking capacity.

### Patient selection and enrolment

Participants will be assessed at the RIE ED majors triage area and those appropriate for the majors area of the ED (including both HD and IC areas) deemed by the treating clinical team will be eligible for inclusion in the study. Participants who are stepped down from resuscitation room care will also be eligible for inclusion in the study.

Potential participants or their relative, guardian or welfare attorney (if lacking capacity) will be approached by a member of the study research team (if a member of the usual care team) or direct care clinician if suitably trained. The potential participants or their relative will receive a copy of the participant information sheet. If the participant has capacity and is agreeable to participate in the study, then informed consent will be taken from the participant. Where a potential participant lacks capacity, informed written consent will be gained from a relative, guardian or welfare attorney.

The study researcher will apply the SNAP40 device and alert the clinical staff in the HD/IC area that the participant has been included in the study. The study team will explain to the clinical team that the study is assessing the performance of the SNAP40 device. If a participant is moved to the HD or IC area of the ED, then a study researcher will monitor the cubicle that they are assigned to. The study team will ensure that study participants are transferred to cubicles whose monitor’s alarms have been set to reflect the NEWS abnormal boundaries for blood pressure, pulse/heart rate, respiratory rate and oxygen saturation. All monitors being used in this study will be checked and reset to reflect NEWS parameters prior to the study starting. It is anticipated that some of the IC participants will be ambulatory within the ED. If such a participant is then deemed to require standard monitoring, the clinical staff will inform the study team and a study researcher will monitor the cubicle. Allowing for departmental workload, the research team will attempt to ensure that any cubicles with a study participant are within vision of the study researcher without changing the participant’s clinical priority categorisation.

### Screening for eligibility

Potentially eligible patients will be identified by medical and nursing staff in the ED and also by the Emergency Medicine Research Group of Edinburgh (EMERGE) researchers (part of the patient’s direct care team) screening in accordance with EMERGE Research Governance: Data protection and Confidentiality SOP current version. Eligibility will be confirmed by the study researcher delegated to the study.

Non-recruited but potentially eligible patients aged 16 years or over, triaged to high dependency (HD)/immediate care (IC) areas will be identified by a search of all ED attendances at the end of the study to estimate the proportion of ED attendances who have participated in the study.

### Treatment allocation

Participants will firstly be allocated a participant study number. Basic demographic data and the participant’s baseline observations will then be collected from each participant at enrolment and recorded in the case report form (CRF). The study researchers will then allocate the participant a SNAP40 device. Each device has a unique ‘identifier’ that will be recorded on the CRF and also on a separate recruitment log. This will allow the clinical vital sign and clinical alert data from each participant to be collected and recorded on the CRF along with the alerts from the SNAP40 device for the same participant. If a new device (e.g. because of a depleted battery) replaces a SNAP40 device already worn by a participant, the unique ID of the new device along with the reason for replacement and time it is assigned will be recorded on the CRF. No patient identifiable information will be entered into the SNAP40 system.

### Study interventions

The SNAP40 device will be placed onto the participant’s arm. The opposite arm will therefore be available for any standard observation recordings in the department at the discretion of the clinical team. The information from the SNAP40 device will be observed by the research team only and will not be used as an alternative to standard care or clinical observations of the participant whilst they are in the ED. The participant will continue to have their vital signs monitored by the clinical team as per standard practice.

The SNAP40 wearable device will continuously monitor participants throughout their stay and transmit three key signals: photoplethysmograph (PPG) waveform using green, red and infrared light; skin temperature and movement. Anonymised SNAP40 monitoring data will be transmitted continuously by the SNAP40 device via a secure Wi-Fi connection to a SNAP40 server located in the ED. The anonymised SNAP40 monitoring data will be transmitted along with the date and time of the observation and the unique device ID. This will allow the anonymised monitoring data to be associated with the participant study number. The anonymised SNAP40 monitoring data will be securely stored with access restricted to the study staff and authorised SNAP40 personnel. This anonymised SNAP40 monitoring data may be used by SNAP40 in future for product development.

Signal processing algorithms developed by SNAP40 will calculate heart rate, respiratory rate, blood pressure and oxygen saturations from the PPG every 2 s. The SNAP40 device measures change in blood pressure. The SNAP40 device will alert the study researcher in real time via a smartphone or tablet application should the participant’s vital sign physiology change from one NEWS category to a higher one in any of pulse/heart rate, respiratory rate, skin temperature or oxygen saturation reading. Physiological changes relating to blood pressure and movement do not relate to the NEWS score. If blood pressure increases or decreases by 15% of the participant’s baseline, the score increases (or decreases) by ‘1’. If movement is monitored by the device as ‘reduced’, the score is increased by ‘1’. The study researcher will record any deterioration alerts on the CRF. The time of each alarm, reason for each alarm (i.e. parameter for which the alarm was triggered) and new individual component NEWS score will be recorded each time the SNAP40 device sends an alarm to the study team.

The participant will be monitored by the device for a maximum of 4 h during their stay in the ED. The device will be removed prior to the participant being discharged from the ED or admitted to the hospital. Participants will be asked to complete a questionnaire to rate their experience of the SNAP40 device and current monitoring.

As well as recording the SNAP40 alarms, the research team will also observe and record standard practice currently being utilised when observing and monitoring the participant’s vital signs. What the researcher records will differ slightly between participants triaged to high dependency (HD) and immediate care (IC). At the end of the study, research nurses who have worked with the SNAP40 device will be asked to complete a questionnaire rating their experience of both SNAP40 and current monitoring.

### Participants who are triaged to HD

The frequency of vital sign recordings will be set at the discretion of the clinical staff and according to the participant’s clinical priority categorisation. Each time the standard alarms on the Mindray monitor sound, the research team will record the reason for alarm (e.g. parameter for which the alarm was triggered) in any of blood pressure, pulse/heart rate, respiratory rate or oxygen saturation reading and new NEWS parameter score. The study researcher will record any such alerts on the CRF. The time taken to respond and the response to these alarms (e.g. silencing alarm, escalation in care) will also be recorded by the research team. Escalation in care will include ED medical or senior nursing staff review, other speciality review, critical care review and movement to the resuscitation bay or to an area of increased priority. Raw data from the Mindray monitors will not be extracted and stored.

Total time spent recording all observations will be noted for at least 25 patients in the study. To do this, the research team will, where possible, time how long clinical staff spend recording each observation on the NEWS chart. If the researcher is unable to time an observation being completed by a staff member, this will be indicated on the CRF. The total number of observations recorded, along with the total number timed and not timed will also be recorded on the CRF.

The NEWS chart recordings noted on the participant’s ED shock chart (i.e. blood pressure, pulse/heart rate, respiratory rate, oxygen saturation reading, core temperature using a tympanic temperature probe and neurological status assessed by GCS) will also be reviewed, and any deterioration in NEWS score will be recorded in the CRF. The time and parameter for which the NEWS score changed will be recorded. A photocopy of the participant’s ED shock chart will be taken prior to them leaving the ED in order to ensure all data is collected and allow it to be checked later on if necessary. This chart will be anonymised with the participant study number and kept with the participant’s study paperwork.

### Participants who are triaged to IC

The frequency of vital sign recordings will be set at the discretion of the clinical staff and according to the participant’s clinical priority categorisation. Each time the participant has their observations taken by a clinical staff member, the research team will record the time taken, and again where possible, total time taken to complete the observation. As above, if the researcher is unable to time an observation being completed by a staff member, this will be noted on the CRF. The total number of observations recorded, along with the total number timed and not timed, will be recorded on the CRF.

Clinical staff response (i.e. nothing done, escalation in care) to a deterioration in NEWS score will also be recorded in the CRF. Escalation in care includes ED medical or senior nursing staff review, other speciality review, critical care review and movement to the resuscitation bay or to an area of increased priority. It is hoped that the researcher will observe, in real time, the response to any deterioration in NEWS and record this on the CRF. However if this is missed, the researcher will retrospectively ask the member of staff taking care of the patient what was done, if anything, about it. A photocopy of the participant’s ED shock chart will be taken prior to them leaving the ED in order to allow this data to ensure all data is collected and allow it to be checked later on if necessary. This chart will be anonymised with the participant study number and kept with the participant’s study paperwork.

The participant’s CHI number, episode number, participant study number and their allocated SNAP40 ‘identifier’ number will be recorded on a separate database held on a secure password protected drive. This database will only be able to be accessed by NHS study staff and will not leave the NHS. This will also ensure that no participant identifiable information is contained with the CRF or electronic CRF.

The SNAP40 device is being used alongside usual care equipment and not instead of it. In this study, the study team will not intervene to inform clinical staff should deterioration in a patient’s vital sign physiology be detected by the SNAP40 device except for the extreme following examples in which case they will intervene as appropriate to the situation:

- Cardiorespiratory arrest
- Blood pressure reading showing increase or decrease
- Pulse/heart rate < 40 or > 200
- Respiratory rate < 5
- Oxygen saturation < 80%

If a standard alarm is not answered by the clinical team and on investigating it is for one of the above reasons, then again the research team will intervene as appropriate to the situation.

The CRF will contain several questions relating to participant and staff satisfaction of both SNAP40 and current monitoring, and participant and staff confidence in the detection of abnormal vital signs in the ED which will be posed to participants and clinical staff involved in the study.

### Trial assessment schedule

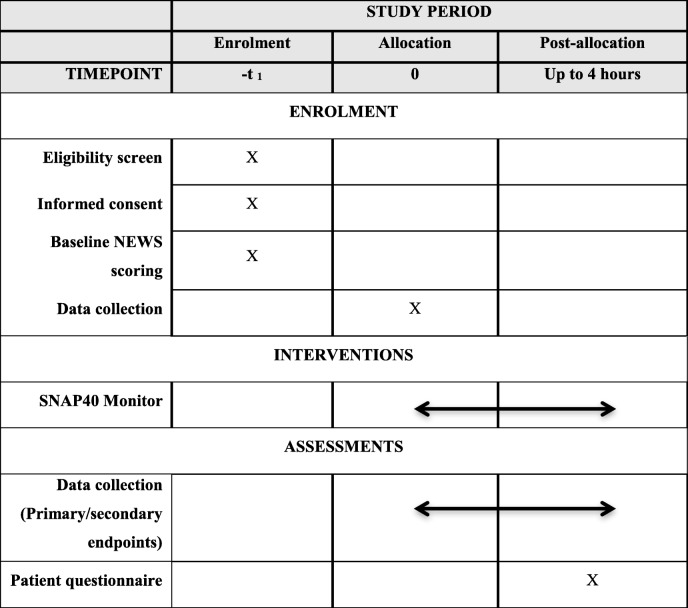



### Participant follow-up

#### Data collection

Participants will have information collected on their CRF during index hospitalisation transferred to a specially designed electronic case report form (eCRF) database. No patient identifiable information will be entered onto this specially designed eCRF database.

#### Statistics and sample size calculation

A small pilot study in the RIE ED demonstrated that 20% of patients experienced deterioration in NEWS score during their ED stay. We aim to recruit 250 patients. Assuming that 20% of them deteriorate, we will have 90% power to detect an effect size of 0.468 and 80% power to detect an effect size of 0.404 (paired *t* test, two sided *p* = 0.05). Appropriate statistical analysis will be performed. A statistical analysis plan will be prepared by the study statistician.

#### Study conduct and protocol amendments

Any changes in research activity, except those necessary to remove an apparent, immediate hazard to the participant in the case of an urgent safety measure, must be reviewed and approved by the chief investigator. Amendments to the protocol must be submitted in writing to the appropriate REC and local R&D for approval prior to participants being enrolled into an amended protocol. A data monitoring committee has not been established.

#### Protocol violations and deviations

Protocol deviations will be recorded in a protocol deviation log, and logs will be submitted to the sponsors every 3 months. Each protocol violation will be reported to the sponsor within 24 h of becoming aware of the violation.

The investigators are responsible for the detection and documentation of adverse events. Participants or their relatives will be instructed to contact the study team at any time after consenting to join the trial if any symptoms develop. All adverse events (AE) that occur after joining the trial must be reported in detail in the case report form (CRF) or AE form. In the case of an AE, the investigators should initiate the appropriate treatment according to their medical judgement. The only AEs and SAEs recorded will be those directly related to the use and application of the SNAP40 device. The sponsor has a legal responsibility to notify the relevant Research Ethics Committee that approved the trial.

The study will be coordinated by a Project Management Group consisting of the CI, co-investigators, statistician and research team. A delegation log will be prepared detailing the responsibilities of each member of staff working on the study. Study monitoring and audit will be the responsibility of the sponsor.

### Confidentiality

All evaluation forms, reports and other records will be identified in a manner designed to maintain participant confidentiality. All paper records including CRFs will be kept in a secure storage area, in this case in a locked filing cabinet in the EMERGE office which has limited access.

The participant’s CHI number, episode number, participant study number and their allocated SNAP40 ‘identifier’ number will be recorded on a separate electronic database held on a secure password protected drive. This database will only be able to be accessed by NHS study staff and will not leave the NHS. This will also ensure that no participant identifiable information is contained with the CRF or electronic CRF.

The anonymised SNAP40 monitoring data will be securely stored with access restricted to the study staff and authorised SNAP40 personnel. This anonymised SNAP40 monitoring data may be used by SNAP40 in future for product development.

Anonymised information collected on the CRF will be entered onto a secure database. Members of the study team including those from the University of Edinburgh’s Clinical Trials Unit will view this. This anonymised information may be made available to other researchers in future. No patient identifiable information will leave NHS Lothian in any form whatsoever.

The CI and study site staff involved with this study may not disclose or use for any purpose other than performance of the study, any data, record or other unpublished, confidential information disclosed to those individuals for the purpose of the study.

### Reporting, publication and notification of results

Ownership of the data arising from this study resides with SNAP40. On completion of the study, the study data will be analysed and a clinical study report will be prepared. The clinical study report may be used for publication and presentation at scientific meetings. The patient information sheet and consent form was reviewed by the EMERGE patient and public involvement group.

## Discussion

The SNAP40-ED study aims to recruit 250 patients. It will be the first study to compare the ability of a novel ambulatory monitoring device to detect deterioration compared to standard care in the ED. It may allow the earlier detection of deterioration in the clinical condition of ED patients and therefore earlier treatment to reverse this deterioration and potentially prevent adverse outcomes.

### Trial status

The trial opened to recruitment on 25 September 2017 and is anticipated to run until February 2018 with trial completion by the end of August 2018. As of November 2017, 50% of the study population was recruited. This is protocol version number 3.0, dated 12/12/2017.

## Abbreviations

AE: Adverse event
CI: Chief investigator
CRF: Case report form
ECG: Electrocardiogram
eCRF: Electronic case report form
ED: Emergency department
EMERGE: Emergency Medicine Research Group of Edinburgh
EPR: Electronic patient record
GCS: Glasgow Coma Scale
HD: High dependency
IC: Immediate care
MET: Medical emergency teams
NCEPOD: National Confidential Enquiry into Patient Outcome and Death
NEWS: National Early Warning Score
PICC: Peripherally inserted central catheter
RIE: Royal Infirmary of Edinburgh
SAE: Serious adverse event
SOP: Standard operating procedure
TMF: Trial master file
UK: United Kingdom
